# Scientific and technological challenges of recombinant egg protein production

**DOI:** 10.1186/s12896-025-01002-3

**Published:** 2025-07-02

**Authors:** Franziska Beck, Philipp Noll, Ute Schweiggert-Weisz, Marius Henkel

**Affiliations:** 1 Cellular Agriculture, TUM School of Life Sciences, Technical University of Munich, Freising, Germany; 2 Plant Proteins and Nutrition, TUM School of Life Sciences, Technical University of Munich, Freising, Germany; 3 Fraunhofer Institute for Process Engineering and Packaging, Freising, Germany

**Keywords:** Precision fermentation, Alternative protein, Recombinant food protein, Functional protein, Microbial food biotechnology, Recombinant egg protein, Cellular agriculture

## Abstract

Eggs are among the most widely consumed and versatile animal-derived foods and are valued for their exceptional nutritional and functional properties. However, conventional egg production is associated with significant environmental, ethical, and health concerns, increasing the global consumer demand for sustainable animal protein. To address these challenges, recombinant egg protein production through precision fermentation has emerged as a promising alternative. Yet, this alternative production path is still in its infancy, and current efforts in research have not yet led to a widespread adoption of recombinant egg protein. This review provides an overview of the bioprocesses used to produce recombinant egg proteins, highlighting their nutritional, bio-functional, and techno-functional significance. The current state of the art of recombinant egg protein production is presented, with a comparison of different microbial expression hosts in terms of suitability and associated challenges. Only six egg proteins were reported to be expressed at laboratory scale, including ovalbumin (3.7 g/L with *Escherichia coli* EcN) and ovomucoid (3.2 g/L with *Komagataella phaffii*). The realization of large-scale production of functional egg proteins remains challenging. These challenges include posttranslational modifications, achieving functionality and cost parity to natural egg proteins, efficient resource bioconversion, and optimizing the bioprocess chain (upstream, bioproduction, and downstream processes). This requires further improvements and research to increase protein titers, space-time yields, and production rates. Nevertheless, recombinant egg protein produced via precision fermentation holds great promise as a functional food ingredient. With further advancements, this approach could contribute to global protein demand, enhance food security, and strengthen food system resilience while providing a more sustainable and ethical alternative to conventional egg production.

**Supplementary Information:**

The online version contains supplementary material available at 10.1186/s12896-025-01002-3.

## Introduction

Egg proteins are a vital source of macronutrients in the human diet because of their balanced amino acid (AA) profile (essential and nonessential) [1], high digestibility (biological value of 0.94 [2]), and high content of minerals and trace elements (0.002–390 mg/100 g), vitamins (0.3–2580 µg/100 g), and antioxidants [3]. They also possess relevant techno-functional properties, including emulsifying, foaming, crystallization control, and clarification capabilities, making them valuable in various food applications, from egg powders to baked goods, frozen products, and beverages [4, 5]. World egg production reached 97 million tons annually in 2023, increasing by 40% compared to 2010 [6], and it is expected to double over the next four decades [7]. This leads to an average amount of egg consumption per head of ~ 12 kg, combining whole and processed eggs. However, increasing food production to ensure food security is challenging and requires an elegant balance between food safety and environmental sustainability [7]. Conventional protein production, including the production of meat, fish, egg, and plant proteins, is currently associated with environmental concerns related to the use of land and water, greenhouse gas (GHG) emissions, and the depletion of resources. To produce egg protein globally, 2.9 to 8.5 kg CO_2_eq/100 g egg protein are released, 4.3 to 8.8 m²year/100 g egg protein of land, and 0.4 to 38.8 m^3^/100 g egg protein from stress-weighted water are used [8]. A total energy consumption of 2 to 2.4 MJ/100 g egg protein produced in one year was reported for the European Union by Dekker et al. (2011) [9]. To put these figures into perspective, the annual amount of carbon dioxide (CO_2_) released is equivalent to 0.6 to 1.8 million passenger cars annually in the USA (4.6 t CO_2_/year/car), 0.87 to 84.5 times the annual water consumption of the United States (0.8 trillion m³) and provide energy for all German private households for 3.9 to 4.7 years (2021). Moreover, approximately 81% of chickens and 86% of eggs worldwide are produced in intensive farming units [10]. This leads to a greater risk of zoonotic diseases and, consequently, a significant reliance on antibiotics in this context [10]. Addressing these concerns through alternative and more sustainable protein production is necessary to guarantee a sustainable, safe, and secure food supply for future generations [11]. The umbrella term alternative proteins (APs) refers to protein sources that substitute conventional animal-derived proteins, including plant-based, single-cell, fermentation, and cultured proteins, providing an AP supply for human consumption without traditional animal agriculture [12]. These APs are derived from various sources, including plants, microbial cultures, cell cultures, algae, and insects [13]. APs are intended to be used and processed to closely replicate the nutritional, functional, and sensory characteristics of original animal protein foods (e.g., meat, egg, milk). Replacing eggs with APs requires an understanding of their functional roles. Whole eggs may be replaced with scrambled eggs, yolk for emulsification (e.g., mayonnaise), or white for foaming (e.g., meringues). Some applications, such as batters for baked goods, demand both functionalities, which are determined by the interfacial properties of the proteins. Given the highly functional nature of egg proteins, careful evaluation is needed to determine how APs can be produced and how similar properties can be achieved. The successful production of APs comes with their individual challenges according to the production host systems. For example, only six egg proteins (Table 2) have been expressed heterologously in microbial hosts under laboratory-scale conditions, yet. Thus, recombinant egg protein production technology is still in the early stages of development, and more research is necessary.

## Natural egg proteins

### The Hen egg: composition, nutritional and functional properties

**Fig. 1:**
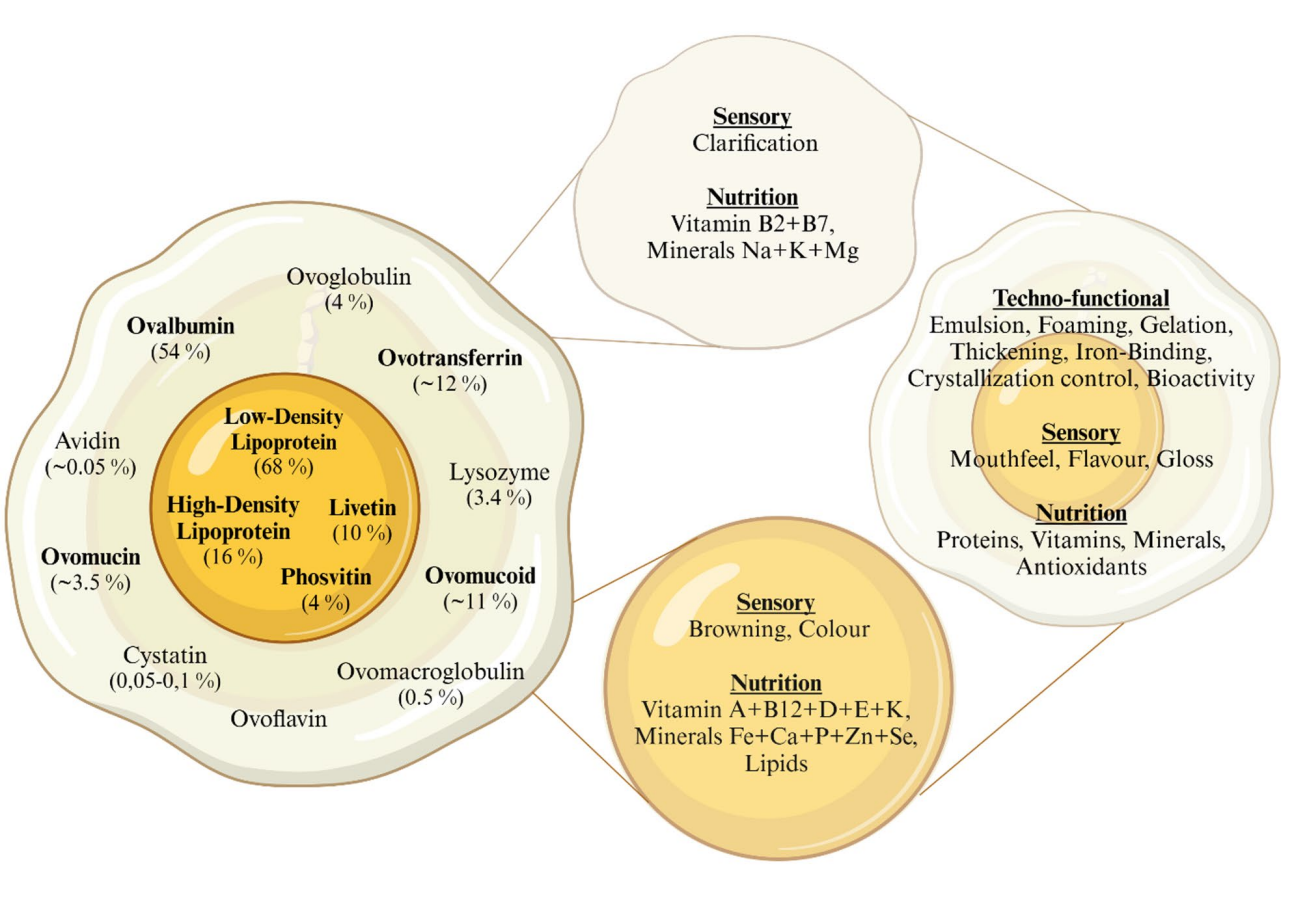
Major and minor egg white and yolk proteins, including their techno-functional, nutritional, and sensory properties. A total of 88.55% (w/w) egg white protein content and 98% (w/w) yolk protein content were related to the total egg white or yolk protein mass (protein properties adapted from [4]) (Created in BioRender. Beck, F. (2025) https://BioRender.com/vkrzkw5)

Hen eggs (*Gallus gallus*) comprise 76.1% (w/w) water, 12.6% protein, 9.5% fat, 1.1% ash, and 0.7% carbohydrate. The macronutrients are distributed throughout the shell, white, yolk, vitelline membrane, and chalazae [3]. Egg white is the primary source of protein, and a total of 808 proteins have been identified in egg white and 813 in the yolk, with 579 unique to egg white, 584 unique to the yolk, and 229 shared. Sarantidi et al. (2023) developed a “Protein Atlas” for egg white and yolk proteins [14]. Eggs are valued for their high-quality, bioavailable protein with a balanced AA composition and high digestibility [15]. They possess important techno-functional properties, such as foaming, emulsifying, and gelling, which are crucial in many food applications [5]. Moreover, numerous biological properties include antioxidant, anti-inflammatory, anticancer, and antimicrobial activities [14]. Figure 1 illustrates primary egg white and egg yolk proteins, along with their mass compositions as well as their techno-functional, sensory, and nutritional properties [4]. An overview of the specific techno-functional, bioactive, and additional properties of egg white and yolk proteins is given in Additional file S1.

### Egg white proteins

The egg white, or albumen, constitutes 58% (v/v) of the total egg and contains primarily water (~ 88% (w/w)) with ~ 11% (w/w) protein and minor amounts of carbohydrates (0.5% (w/w)), lipids (0.2% (w/w)), vitamins, and ash (0.8% (w/w)) [3, 16]. Some egg white proteins, such as ovalbumin, ovotransferrin, ovomucoid, and lysozyme, can cause allergic reactions [17]. The major proteins are ovalbumin, ovotransferrin, ovomucoid, and ovomucin, constituting 80.5% (w/w) of the total protein in egg white. The detailed protein structures and physicochemical properties are shown in Table 1.

**Ovalbumin** (OVA) is a phosphorylated glycoprotein constituting 54% (w/w) of the egg white protein fraction [18]. Although it belongs to the serpin family of protease inhibitors, it lacks inhibitory activity, distinguishing it from other serpin-like proteins [19]. OVA exists in three forms, A1-, A2-, and A3-OVA, distinguished by their phosphate group counts, with the predominant dephosphorylated form [19, 20]. OVA features a unique AA composition with 50% hydrophobic and 30% acidic residues, six cysteine residues (Cys74 and Cys121 form a disulfide bond, and the rest are free of sulfhydryl groups), and distinct structural properties, such as an acetylated glycine at the N-terminus [19]. When subjected to higher temperatures (> 25 °C) and different pH conditions (> pH 9.0), OVA undergoes conversion to a thermally stable variant, designated S-ovalbumin. This transformation impacts the texture and structure of food products, e.g., baked goods [21]. OVA has great functional properties, including foaming, gelling, and emulsification. Moreover, it exhibits antioxidative activity and contains peptide fragments with antimicrobial peptides effective against, e.g., *Bacillus subtilis* (*B. subtilis*), when exposed to proteolytic cleavage during digestion [22, 23]. Previous studies have demonstrated the successful recombinant production of OVA, with Liu et al. (2024) achieving a maximum titer of 3.7 g/L in *Escherichia coli* (*E. coli*) (EcN) (Table 2). **Ovotransferrin** (OVT) is a monomeric glycoprotein with iron-binding properties, constituting ~ 12% of egg white protein [25]. It shares structural similarities with mammalian transferrin, including two N- and C-terminal domains, each with a single iron-binding site [26]. They are connected by 15 disulfide bridges and noncovalent hydrophobic interactions, maintaining their globular conformation [27]. The biological function of OVT is the binding and transport of diverse metal ions, including Fe, Cu, and Zn (naturally important for embryo development) [22]. Disulfide bonds, particularly those between Ala1 and Tyr72, are critical for iron acquisition [28]. Iron is bound at pH values above 7 and released below pH 4.5 [5]. Therefore, OVT could be used as a supplement to deliver bioavailable iron and address the global undersupply of this micronutrient, especially in women of childbearing age [1]. Moreover, it exhibits antimicrobial properties by sequestering iron and inhibiting iron-dependent microbes such as *Pseudomonas* sp., *E. coli*, and *B. cereus* [29, 30]. OVT also reportedly has antioxidant [31], antiviral [32], antifungal (against the genus *Candida*), anticancer [33], and immunomodulatory characteristics [30]. Two articles previously described the successful recombinant production of OVT, with Mizutani et al. (2003) achieving a maximum titer of 97 mg/L in *Komagataella phaffii* (*K. phaffii*) (KM71) (Table 2). **Ovomucoid** (OVM) is a glycoprotein that constitutes ~ 11% of egg white protein and contains sialic acid, ~ 4.5% mannose, and ~ 1.5% galactose [22]. It consists of 186 AAs, with nine disulfide bonds and no free sulfhydryl groups [35]. Owing to its high degree of glycosylation, OVM plays a key role in foaming and emulsification processes [36]. It also has antioxidant [3] and tumor-suppressive properties, highlighting its potential as an anticancer agent [11]. However, OVM is a major allergen implicated in IgE-mediated allergic reactions [18, 37]. Two articles previously reported the successful recombinant production of OVM, with the EVERY Company [38] achieving a maximum titer of 3.2 g/L in *K. phaffii* (Table 2). **Ovomucin** (OVN) is a sulfated glycoprotein constituting ~ 3.5% of the total albumen protein content and is responsible for the gel texture of egg white [18, 39]. It consists of two subunits linked by disulfide bonds: the α-subunit (220 kDa), with 10–15% carbohydrates, which is rich in glutamic acid and aspartic acid, and the β-subunit (400 kDa), which contains 50–65% carbohydrates, primarily serine and threonine [40]. OVN is characterized by a high degree of N-glycosylation [41]. OVN has been reported to exhibit foaming, foam stabilizing, and emulsifying characteristics, whereas peptides derived from OVN have antiviral and antitumor effects [42]. OVN also degrades over time, impacting egg white thinning during storage, potentially through disruption of the OVN-LYS complex or reduction in disulfide bonds [43]. Until the time of submission of this article, no recombinant production of OVN was known to the authors.

There are several **minor proteins** in egg white; however, six are important for the physicochemical and structural properties of the egg. An additional table file shows the different functional activities of each egg white protein in more detail [see Additional file S1]. **Lysozyme** (LYS) is a highly soluble, stable monomer constituting 3.4% of the egg white protein [18, 39]. LYS has two domains: hydrophobic properties at its core and hydrophilic properties at its surface [44]. It can form dimers with negatively charged proteins (e.g., OVA, OVN, and OVT) in egg whites [43]. As a bactericide, LYS hydrolyzes the β [1–4] glycosidic bond in bacterial peptidoglycan, making it effective against gram-positive bacteria [44]. Two articles previously reported the successful recombinant production of LYS, Cui et al. (2022) reported a maximum titer of 3.5 g/L in *K. phaffii* (GS115) (Table 2). **Ovoglobulins** constitute 4% of egg white proteins and comprise two subunits (G2: 36 kDa and G3: 45 kDa), N-glycosylated and O-glycosylated [18]. It plays a key role in egg white foaming and coagulation, even though other biological functions are poorly understood [18]. **Cystatin** constitutes ~ 0.05–0.1% of egg white protein and can appear in a phosphorylated (pI 5.6) or nonphosphorylated form (pI 6.5) [5, 18]. Cystatin contains up to two disulfide bonds and a highly conserved reactive site with a cysteine proteinase inhibitor (which inhibits thiol proteases) [18, 46]. It has several bioactive properties. **Ovomacroglobulin** (ovostatin) constitutes 0.5% of egg white protein. It comprises four identical glycosylated subunits, two of which are disulfide-bonded. It acts as a protease inhibitor by trapping enzymes within its structure and has diverse bioactive properties (Table S1) [47, 48]. **Avidin** is a tetrameric glycoprotein constituting ~ 0.05% of egg white protein and is known for its strong biotin-binding ability (vitamins B7 and H) in all four inner regions [22, 39]. It forms a highly stable avidin-biotin complex used in various applications, e.g., enzyme-linked immunosorbent assays, and offers bioactive properties [22]. Two articles previously reported on the successful recombinant production of avidin. Zocchi et al. (2003) reported a maximum titer of 330 mg/L in *K. phaffii* (GS115) (Table 2). **Ovoflavin** (riboflavin-binding protein) is a phosphoglycoprotein found equally in egg white and yolk. It is a pyroglutamic acid at the amino terminus, and N-linked glycosylation and phosphorylation are crucial for riboflavin binding and transport. Among egg proteins, egg proteins have the highest bioavailable selenium content (1.8 µg/g) [18, 49]. Until the time of submission of this article, no recombinant production of ovoglobulin, ovomacroglobulin, or ovoflavin was known to the authors.

### Egg yolk proteins

Egg yolk (EY) is a complex matrix constituting 31% of the egg and contains 50% water, 31–35% lipids, 15–17% proteins, 0.6–1% carbohydrates, and 1.7% minerals all in (w/w) [16, 39]. Hen EY consists of 19–23% (w/w) insoluble egg yolk granules (EYGs) rich in high-density lipoproteins (HDLs) and phosvitins and 77–81% (w/w) egg yolk plasma (EYP) containing low-density lipoproteins (LDLs) and livetins [3]. EYP accounts for ~ 50% of the EY proteins and 90% of the EY lipids, comprising 85% of the LDLs and 15% of the liveetins. EYGs represent the remaining ~ 50% of the total EY proteins and 7% of the EY lipids, containing ~ 70% HDLs, 16% phosvitin, and 12% LDLs. EYP and EYGs exhibit emulsifying, film-forming, and antioxidant properties [50]. An additional table file shows the different functional activities of each major EY protein in more detail [see Additional file S1]. EY proteins possess amphiphilic side chains that stabilize emulsions by adsorbing to oil‒water interfaces. Dissociated EYGs, under high salt (NaCl 0.55 mol/L) or pH conditions (pH 7.0), increase emulsion stability because of the solubilization of HDLs and phosvitin [51]. EY also shows potential in preventing pathogen colonization (e.g., *Salmonella*, *E. coli*, *Campylobacter*), reducing egg contamination [52]. However, the complex mix of EY components makes precise control of their physical properties challenging, necessitating further research [53]. Moreover, EY exhibits low-level allergenicity, e.g., as mediated by R-livetin [17]. Minor EY proteins, such as lipovitellin and yolk LYS, comprising ~ 2% of total EY proteins, may still contribute to the functional and nutritional properties of EY but are not detailed in this review.

**Low-density lipoproteins** are glycosylated and phosphorylated and constitute the predominant protein component of EY, accounting for 68% of its total protein content [3]. Primarily located in yolk plasma, LDLs have a micellar nanostructure with a triglyceride, cholesterol, and cholesteryl ester core surrounded by apoproteins and phospholipids [50, 54]. The LDLs comprised 11–17% protein and 83–89% lipids, with 74% neutral lipids and 26% phospholipids. Phospholipids maintain structural integrity via hydrophobic interactions [54, 55]. Their key technique is emulsification, which is facilitated by the absorption of apolipoproteins at oil-water interfaces [18, 53]. Protein functionality is affected by factors such as ionic strength, pH, heat, and defatting [56]. Until the time of submission of this article, no recombinant production of LDL was known to the authors. This may be due to the large size of LDL and their need for co-translational lipidation of apolipoprotein components. In addition, a precise assembly of phospholipids and neutral lipids is required, which exceeds the capacity of standard prokaryotic and yeast secretion pathways. Furthermore, LDLs require the formation of large complexes for proper folding, which makes them insoluble when expressed individually [18]. **High-density lipoproteins** constitute 16% of EY protein [3]. In yolk granules, HDL forms insoluble complexes with phospholipids, primarily phosvitin. This creates hydrophobic cavities capable of binding up to 40 bioactive compounds (e.g., phospholipids) [51, 57]. At low ionic strengths (< 0.17 mol/L NaCl), HDL is insoluble and bound to phosvitin [51]. At higher ionic strengths (> 0.3 mol/L NaCl), HDL and phosvitin dissociate, enhancing emulsifying properties [58]. HDL also exhibits antioxidant activity and protection against pathogens such as *Salmonella enteritidis* and *E. coli* O157:H7 [18, 52]. Until the time of submission of this article, no recombinant HDLs were known to the authors. The lack of literature on recombinant HDLs might have similar reasons like the lack of reports on LDLs. HDLs like LDLs have a complex and large structure, include lipids, and the replication of their functional properties are therefore challenging. Relevant native eukaryotic systems, such as ABCA1-mediated lipidation and posttranslational modifications, may be necessary [59]. **Phosvitin** is a highly phosphorylated glycoprotein comprising 4% of total EY proteins [3]. It is composed of 9.7% phosphorus, 11.9% nitrogen, and 0.7% lipids and consists of two polypeptides, α-phosvitin (160 kDa) and β-phosvitin (190 kDa) [60, 61]. Phosvitin contains 124 of 217 AAs that are covalently bound to phosphate. Approximately 50% of the AAs in phosvitin are serines, of which up to 90% are phosphorylated [61, 62]. Because phosvitin has a high negative charge due to the extensive phosphorylation of serine residues, it has strong metal-binding properties, particularly for iron (which binds ~ 95% of yolk iron) [60, 63]. The negatively charged phosphate groups form stable chelat-complexes with divalent and trivalent cations (e.g., Ca²⁺ and Fe³⁺). Adjacent phosphoserine clusters allow for cooperative multivalent binding, which reduces entropic penalties and enhances overall affinity [64]. The iron-binding capacity of phosvitin enables several biological functions. The short hydrophobic C-terminal region of phosvitin is relevant for its amphiphilic character, enabling emulsion-stabilizing properties [50, 65]. Until the time of submission of this article, no recombinant production of phosvitin was known. **Livetin** proteins are water-soluble glycoproteins constituting 10% of EY protein and appear in three forms: α-livetin (albumin), ß-livetin, and γ-livetin [3, 18]. Livetin has high solubility (> 86% in distilled water), foaming capacity (21–58%), and emulsification activity (7.3–9.7 m^2^/g) across a wide pH range [2–12, 66]. Only one paper has previously described the successful recombinant production of α-livetin, with De Silva et al. (2018) achieving a maximum titer of 54 µg/L in *Kluyveromyces lactis* (GG799) (Table 2) [67].

**Table 1:** Physicochemical properties of egg white and yolk proteins

Protein	Molecular weight [kDa]	Amino acids	Isoelectric point (pI)	Denaturation Temperature (T_d_) [°C]	Di-sulfide bonds	Posttranslational modifications	Genetic Polymorphism (position)	Allergenicity	References
**Major egg white proteins**									
Ovalbumin	45	386	4.5–4.7	84	1 (Cys74–Cys121)	• Non, one or two phosphorylations at serines 69 and 345 • Acetylation (glycine at N-termini and proline at C-termini) • Glycosylation	Glu→Gln (290) and Asn→Asp (312)	Yes, primary	[16–19]
Ovotransferrin	76-77.7	686	6.0-6.1	61	15 (Ala1–Tyr72)	• N-glycosylated	No	Yes	[16–18, 25, 27, 28]
Ovomucoid	28	186	4.1	77	9	• N-glycosylated	No	Yes	[17, 18, 68]
Ovomucin	132	1185	4.5-5.0	n.d.	8	• N- and O-glycosylated • Proteolytic Cleavage	No	No	[16, 18, 41] Uniprot-F1NBL0
**Major egg yolk proteins**									
LDL	130	n.d.	n.d.	n.d.	n.d.	• O-glycosylated • Phosphorylated • Lipid modifications	No	No	[3, 18, 54]
HDL	n.d.	n.d.	n.d.	n.d.	n.d.	• Glycosylation • Lipid modifications	No	No	[18, 69]
Phosvitin	35	217	4.3	n.d.	Non	• Phosphorylation • Glycosylation	No	No	[18, 62, 70] Uniprot-P02845
Livetin	α-livetin: 80 ß-livetin: 45 γ-livetin: 170	α-livetin: 615	5.7	83.3	17	• N- and O-glycosylated	No	Yes, α- and γ-livetin	[17, 48, 66] Uniprot-P19121
**Minor egg white proteins**									
Lysozyme	14.4	129	10.7–11.0	75	4	• N-glycosylated	No	Yes	[16–18, 71, 72]
Ovoglobulin	G2: 36 G3: 45	G2-unit: 439	5.5–5.8	92.5	Non	• N- and O-glycosylated	Yes, only for G2 subunit	No	[16, 18] Uniprot-I0J170
Cystatin	13-15.3	139	5.6 (phosphorylated), 6.5 (non-phosphorylated)	n.d.	2	• N- and O-glycosylated	No	No	[5, 16, 18, 46] Uniprot-P01038
Ovomacroglobulin	166	1437	4.5	n.d.	Non	• N-glycosylated • Proteolytic cleavage	No	No	[18, 47, 48] Uniprot-P20740
Avidin	16.7	152	10	n.d.	1	• N-glycosylated	No	No	[16, 18, 22] Uniprot-P02701
Ovoflavin	27.2	238	4.0	n.d.	8	• N-glycosylated • Phosphorylated	No	No	[18, 49] Uniprot-P02752

‘n.d.‘ represents not defined

## Alternatives to animal-derived egg proteins

### Plant-based egg proteins

Among the alternatives to egg or egg proteins, plant proteins have been the most extensively studied. Plant-based protein ingredients, i.e., isolates, concentrates, and flours, vary widely in protein content and techno-functional properties [73]. In particular, the chemical composition of these ingredients makes it challenging to replace eggs in terms of nutritional quality [74]. Protein flours and concentrates contain carbohydrates that are not included in eggs, and the protein composition itself also varies, as plant proteins usually do not contain the full complement of indispensable AAs, which egg protein does. In addition, the presence of antinutritive substances, such as trypsin inhibitors, which impair protein digestion; phytic acid, which can complex multivalent cations and affect their absorption; or phenolic compounds, which can impair the color of the products [75], is also important. In recent years, several plant proteins, including those from soy, potato, pea, and mung bean, have been extensively studied in recent years. A comprehensive description of the available protein-rich raw materials is provided in Viana et al. (2023) [76]. However, despite significant research efforts, few of these proteins are commercially available as ingredients. This is largely due to the challenges of scaling up processing and isolation. While these challenges differ from those in recombinant protein production, they still pose significant hurdles. As a result, soy protein remains the dominant plant protein on the market, offering high functionality, including excellent emulsifying, gelling, and foaming properties, which can vary slightly depending on processing. While versatile, soy protein has several drawbacks, such as its poor reputation for deforestation issues [77], its ability to be a genetically modified organism (GMO) [78], and its status as an allergen [79]. Peas, which are increasingly entering the market, offer advantages because of their low allergenic potential and because they do not need labeled allergens. Studies have shown that they can mimic eggs’ foaming and texturing properties, especially in baked products [80]. However, improvements are needed because of their poor solubility and poor sensory properties [73, 81]. Finally, mung bean protein isolate, which was approved as a novel food in the European Union in 2023, has already been successfully used in plant-based scrambled eggs and similar products. Ongoing research aims to optimize its functional properties and cost efficiency [82, 83]. Despite these advances, replicating the multifunctionality of eggs remains challenging. Additionally, modification techniques such as enzymatic hydrolysis, which improve the foaming properties of legume proteins, are being explored [84, 85]. The development of blends of different plant proteins or combinations of proteins and hydrocolloids is also being investigated to more closely mimic the functionalities of eggs [86].

### Precision fermentation

An alternative approach to achieve egg-like functionality is the use of recombinant protein technology to produce bioidentical egg proteins. In the recent past, the term precision fermentation (PF) was created to describe the application of microbial hosts as “cell factories” to generate specific functional ingredients, such as egg proteins [87]. This technology offers a potentially more sustainable and scalable alternative to traditional egg protein sources, reducing reliance on animal agriculture while maintaining the functional properties required for food applications. Suppose these proteins are nature-equivalent and free of nucleic acids from the production organism. In that case, it can be assumed that nature-identical proteins will have advantages regarding regulatory approval and consumer acceptance. The technology behind recombinant protein production, such as recombinant insulin and industrial enzymes such as proteases, lipases, and glycoside hydrolases (e.g., recombinant bovine chymosin, Lipopan F, and alpha-amylases), has been established for decades [88]. A new demand that APs pose for PF is that these proteins need to be produced in bulk (> 50 g/L [89]) and at competitive costs. Furthermore, they have a new intended use in the end product, meaning that they are major ingredients rather than just additives used in upstream processing [90]. PF offers advantages over plant-based proteins by mimicking animal proteins and their techno-functional, nutritional, and sensory properties. Unlike plant-based alternatives, recombinant proteins can replicate unique properties such as foaming, emulsifying, and water binding [11, 91]. Furthermore, PF provides environmental benefits, with a recently published life cycle assessment (LCA) indicating up to 90% reductions in land use and 31–72% reductions in GHG emissions compared with conventional egg production, particularly when a full supply of renewable energy is used [92]. Combining bioidentical recombinant protein production with a reduced environmental footprint makes PF a promising technology for sustainable protein production.

### State of the art of Recombinant egg protein production

 A few companies and academic teams have been established worldwide that target recombinant egg protein production via bacteria, yeast, and fungi (Table  2). Most studies have focused on allergenicity and protein biochemistry (e.g., OVM and OVA in *E. coli*). To date, no optimized bioprocess for prokaryotic systems has been developed to produce egg proteins for human consumption. Furthermore, reports on the recombinant expression of EY proteins have been limited to α-livetin (Table  2). In the United States, the EVERY Company pioneered the commercial production of recombinant egg white proteins, launching “EVERY EggWhite” in 2022. This product contains the recombinant egg white protein OVM (patent filed in 2016 [38]) produced by *K. phaffii*. EVERY EggWhite has received a “no questions” letter from the FDA (Food and Drug Administration) [93] and is currently available for bakery and beverage applications only in the US market. It has been reported that EVERY egg whites mimics the functional properties of natural egg whites [11, 94, 95]. Collaborations in 2022 targeted the use of egg whites containing bakery products (Chantal Guillon “Every EggWhite Macarons”, San Francisco, CA, USA) [96, 97] and protein-rich lifestyle drinks (Pulp Culture “Hard Juice”, $16.99/pack of 4, Santa Monica, CA, USA) [98]. These findings highlight the first commercialization attempts for recombinant egg white proteins; however, they were only promotional campaigns and did not have a lasting impact on the consumer market. In a database maintained by the Good Food Institute [99], only four companies are listed that focus on nature-identical egg white proteins (Eggmented, Otro, EVERY, Anthology). The Onego Bio Company, which produces recombinant Bioalbumen ^®^ via *Trichoderma reesei* [100], and the protein brewery company, which produces recombinant OVA in *Aspergillus niger*, were not included in this list at that time [101]. Even though significant investments have been granted to some companies (EVERY $239.8 M [89], Onego Bio $70.8 M [102]), widespread adoption of recombinant egg proteins in a market-ready product still needs to be achieved. Despite these investments, no company has yet been able to establish a large-scale market presence or bring a product to sustainable commercial success.

**Table 2:** Recombinant expression of egg proteins as described in the scientific literature and by companies. Unless otherwise stated, cultivation was performed in shake flasks

Organism	Species	Product	Plasmid	Promotor	Titer and Duration	Intra / Extra cellular	Soluble / Inclusion bodies (IBs)	Quantification method	Reference
Bacteria	*E. coli* (EcN)	Ovalbumin	pMUT2	P_tac−P5_	42 mg/L (24 h); 3.7 g/L (after 47 h in 3 L bioreactor)	Intra	Soluble	Relative fluorescence intensity measurement, SDS-Page	[24]
	*E. coli* (BL21(DE3))	Ovalbumin	pET3d	T7lac	70 mg/L (IBs), 66 mg/L (soluble) (3 h after induction)	Intra	IBs and soluble	SDS-Page	[103]
	*E. coli* (JM109)	Ovalbumin	pKK233-2	ptrc	0.08 mg/L (16 h)	Intra	Soluble	SDS-Page	[104]
	*E. coli* (JM109)	Ovalbumin	pUK233-2	ptrc	0.64 mg/L (16 h)				
	*E. coli* (BL21(DE3))	Ovalbumin	pET3d	T7	84.5 mg/L (16 h)				
	*E. coli* (BL21 (DE3))	Ovotransferrin	pET3d	T7	4 mg/L (16 h)	Intra (in cytoplasm and periplasm)	Soluble	SDS-Page, Western blot	[105]
	*E. coli* (BL21(DE3))	Ovotransferrin	pET20b	T7	100 µg/L (16 h)				
	*E. coli* (M15)	Ovomucoid	pREP4	T5	~ 4–5 mg/L (4 h; after purification)	Extra	Soluble	SDS-Page, Western blot	[106]
	*E. coli* (JM109)	Avidin	pAVEX8	trc-lacI^q^	~ 0,25 − 0,5 mg/L (4 h)	Intra	IBs (at 37 °C) and soluble (at 24 °C)	SDS-Page, Western blot	[107]
	*E. coli* (JM109)	Avidin	pAVEX15	trc-lacI^q^	> 1 mg/L (4 h)				
Yeast	*K. phaffii* (X-33)	Ovalbumin	pGAPZαA	GAP	~ 10 mg/L (2 days)	Extra	Soluble	SDS-Page, Western blot	[108]
	*K. phaffii* (X-33)	Ovalbumin	pGAPZαA	GAP	n.d.	Extra	Soluble	SDS-Page, Western blot	[109]
	*S. cerevisiae* (W303-1b, W303-1b ΔCNE1)	Ovalbumin	pRS426	T7-T3					
	*K. phaffii* (KM71)	Ovotransferrin	pPIC3.5/oTf	AOX1	97 mg/L (4–6 days after induction)	Extra	Soluble	SDS-Page, Western blot	[34]
	*Komagataella phaffii*	Ovomucoid	N/A	AOX1	3.2 g/L (168 h)	Extra	Soluble	SDS-Page, Western blot	The EVERY Company [38]
	*K. phaffii*	Ovomucoid	pGAPZαA	GAP	1 g/L (4–6 days)				
	*Kluyveromyces lactis* (GG799)	α-livetin	pKLAC2	Lac4	54 mg/L (6 days)	Extra	Soluble	SDS-Page, Western blot	[67]
	*K. phaffii* (GS115)	Egg white lysozyme	pPIC9K	AOX1	3.5 g/L (168 h after induction in 15 L bioreactor)	Extra	Soluble	SDS-Page	[45]
	*K. phaffii* (X-33)	Egg white lysozyme	pPIC6α	AOX1	400 mg/L (100 h after induction in 3 L bioreactor)	Extra	Soluble	SDS-Page, Western blot	[110]
	*K. phaffii* (GS115)	Avidin	pPIC9K	AOX1	330 mg/L (65 h after Induction in 3.6 L bioreactor)	Extra	Soluble	SDS-Page, Western blot	[111]
Fungi	*Trichoderma reesei* (M1908)	Ovalbumin	pAWP145	synthetic	2 g/L (≥ 72 h in 1 L bioreactor)	Extra	Soluble	SDS-Page	[91]

‘n.d.‘ represents not defined

## Challenges of recombinant egg protein production

As outlined previously, recombinant egg protein production via PF potentially offers significant advantages over both conventional and plant-based egg substitutes. However, this technology generally still faces certain challenges regarding scalability and efficiency, which will be discussed. An overview of the challenges and potential strategies for advancing recombinant egg protein production is given in Table 3.

**Table 3:** Challenges and potential strategies for advancing recombinant egg protein production

	Challenge	Potential solution	Egg proteins	References
Strain development	**Translational efficiency**	Codon optimization (Codon bias, tRNA drain (repetitive codons), Sec-pathway (rare codons) for decelerated transcription), Promoter engineering, ribosome binding sites (secondary mRNA structure, temperature)	ovalbumin (rich in AT), ovotransferrin (rich in GC), phosvitin (requirement of rare tRNA for phosphoserine)	[11, 90, 112–114]
	**Secretion**	Sec Pathway (periplasmic leader sequences (e.g. pelB, ompA, ompT), secretion factors (e.g. secE, secY)), ESETEC^®^ system	n.d.	[113, 115]
	**Fusion Tags**	Increasing solubility (MBP), simplified purification (His-Tag)	For hydrophobic egg proteins (e.g. lipovitellin, phosvitin), avoiding aggregation and IBs	[3, 113, 116]
	**Transcription factors**	Co-Expression	(bovine lactoferrin with 4.3-fold) yield)	[90]
	**Proteolytic degradation**	Deletion of proteases (*ion* and *OmpT*), pH and temperature regulation, protease inhibitors	e.g. ovalbumin (lacks protective structural features, e.g. disulfide-bonds or extensive phosphorylation)	[91, 113]
PTMs	**Phosphorylation**	*E. coli* B95(DE3) ΔA ΔfabR ΔserB, *E. coli* BL21 ΔserB (serine phosphorylation)	phosvitin (90% of serines are phosphorylated)	[114, 117]
	**Disulfide bridges**	Chaperones (Dsb system), trxB/gor mutants (Origami & Rosetta-gami system), signal peptide periplasm	e.g. ovotransferrin (15 bridges), ovomucoid (9 bridges)	[90, 113, 118]
	**Glycosylation**	*N*-glycosyltransferase system, *ΔwaaL* mutation in *E. coli*, GlycoSwitch^®^ yeast strains	most egg proteins	[119–121]
Production	**Foam**	Chemical antifoam agents (e.g. dimethicone), mechanical foam-control systems))	e.g. ovalbumin	[90, 122, 123]
	**DSP**	Precipitation, adsorption-based methods, aqueous two-phase systems, magnetic separation, (chromatography) etc.	successful purification of e.g. ovotransferrin, phosvitin, ovalbumin, lysozyme, avidin	[124–129]
	**Protein stability and functionality**	Optimal parameters for protein half-life and storage (e.g. pH, ionic strength, temperature and proteolytic degradation), protein formulation (e.g. spray drying)	n.d.	[130]

### Host selection and strain development

Currently, various **host organisms** serve as “workhorses” for industrial biotechnology and PF, including bacteria (e.g., *E. coli* and *B. subtilis*), yeasts (e.g., *Saccharomyces* sp. and *Komagataella* sp.), and filamentous fungi (e.g., *Trichoderma reesei* and *Aspergillus niger*) [36, 122]. Typically, the product and its intended application (food, pharmaceutical, chemical) determine the choice of the host system as well as the upstream, bioproduction, and downstream processes (DSP) [116]. In addition to the common optimization and process design goals of industrial biotechnology, high productivity (titer, rates, yields), process scalability, robustness, and reproducibility, other factors are relevant for producing recombinant egg proteins. These include food safety and the functional characteristics of the product, which are mediated by posttranslational modifications (PTMs) [122]. An additional table file shows the advantages and disadvantages of each expression system in more detail [see Additional file S2].

**Bacteria**, particularly *E. coli*, are the preferred host for egg protein production because of their high theoretical productivity, high growth rate (short doubling time of 30 min), high cell density (maximum theoretical concentration of 160–200 g/L dry cell weight [131]), recombinant protein yield of up to 50% total protein (0.50–0.54 g/g glucose [132]) [133], genetic accessibility, low media requirements and established genetic engineering techniques [120, 122]. For food applications, high volumetric productivity is critical to ensure cost-effective production. To date, *E. coli* has been used for the recombinant production of OVA, OVT, OVM, and avidin (Table 2). Engineered *E. coli* strains such as BL21(DE3) have been optimized through protease deletions (e.g., Lon and OmpT) and advanced expression systems to improve the stability, yield, and solubility of recombinant proteins. Fine-tuning is achieved via both positively regulated promoters (e.g., arabinose or rhamnose operons) and negatively regulated systems (e.g., T5- and T7-based systems with lac or tac promoters induced by IPTG or lactose) [122]. Challenges for recombinant egg protein production with *E. coli* include limited secretion capabilities, disulfide bond formation, PTMs, and inclusion body (IB) formation (cf. Section “Post-translational processing and modifications of protein functions”). Despite these limitations, *E. coli* is well suited for proteins that do not necessarily require PTMs. Since it is not entirely clear if or in which cases PTMs are required for techno-functionality in food applications, bacteria such as *E. coli* remain a cost-effective, scalable option for egg protein production. **Yeast** expression systems offer an attractive alternative when egg proteins require more specific PTMs, as their expression system better supports this approach [90]. These proteins can perform N-glycosylation and phosphorylation, which are critical for the proper folding and functionality of certain egg proteins, such as phosvitin. *K. phaffii* (formerly *Pichia pastoris*) is favored because of its fully sequenced genome, ability to grow to very high cell densities (up to 150 g/L dry cell weight), relatively short doubling time (60–120 min), and high-level protein expression (up to 15 g/L; intra- or extracellular level) [119, 120]. The strong AOX1 promoter in *K. phaffii* allows methanol-inducible expression. However, using methanol on a large scale is unfavorable because of its toxicity [122]. *K. phaffii* has been used to produce recombinant OVA, OVT, OVM, α-livetin, egg white LYS and avidin (Table 2). However, further research is needed to improve promoter systems, and genetic stability establishes induction systems suitable for food proteins and protein degradation. **Filamentous fungi**, such as *Aspergillus niger* and *Trichoderma reesei*, are known for their high secretion efficiency, ability to perform complex PTMs and ability to use lignocellulose, an inexpensive and abundant substrate [11]. However, fungi face limitations compared with bacteria or yeasts, such as lower growth rates (doubling time ~ 2–3 h), lower cell density (up to ~ 30 g/L CDW), low recombinant protein titers (up to 3 g/L) and challenges in genetic manipulation. This is due to their robust cell walls and multicellular morphology, which makes them less widely used for recombinant egg protein production than bacterial and yeast systems. However, their natural ability to secrete large amounts of protein could be exploited for specific applications and to overcome secretion issues [122].

The production of recombinant egg proteins requires optimizing genetic modifications and expression strategies to improve yield, folding, and stability (Table 3). While the following approaches focus on laboratory-scale production, they are transferable to industrial strains tailored for specific recombinant egg proteins. One major hurdle is low **translation efficiency**, especially for AT- or GC-rich genes, as in the case of OVA (~ 60% AT and 40% GC (UniProt P01012)) and OVT (~ 40–45% AT and ~ 55–60% GC (UniProt P02789)). Codon optimization helps to overcome this by matching host-specific codon usage with available tRNAs, thereby improving protein expression [11, 90]. In addition, genetically engineered strains such as BL21-CodonPlus (DE3) RIPL/RIL/RP and Rosetta-gami provide rare tRNAs, improving the production of full-length functional proteins [113, 134], e.g., for phosvitin requiring specific tRNA^Sep^ for phosphoserines [114]. Further yield improvements can be achieved through promoter engineering, where combinations such as lacUV5 and lac promoters have been shown to increase recombinant protein levels in *E. coli* more than tenfold [90]. The optimization of ribosome binding sites is crucial for translation initiation, especially for complex proteins such as OVT. mRNA secondary structures near the RBS mask key elements (the Shine-Dalgarno sequence and the start codon) and hinder ribosome binding. Higher temperatures help destabilize these structures and improve access, while lower temperatures stabilize them and reduce initiation efficiency [112, 113]. Extracellular **secretion** is often not possible or inefficient, which in most cases complicates the DSP. To overcome this, periplasmic leader sequences (e.g., pelB, ompA, ompT) and the coexpression of secretion factors (e.g., secE, secY) facilitate extracellular protein release in *E. coli* [113]. Using a microbial system for increased secretion, such as the proprietary ESETEC^®^ system [115], is particularly beneficial for proteins such as OVT, which require proper secretion for correct folding. Aggregation and poor solubility are major challenges, especially for EYG proteins (HDL and highly phosphorylated phosvitin) [3]. **Fusion tags**, small peptides or proteins linked to the N-terminus of recombinant proteins, are often used in bacterial systems (less commonly in yeast) to increase the solubility, stability, and purification of recombinant proteins [90]. Common tags established in a laboratory environment, such as polyhistidine (6xHis) and glutathione S-transferase, allow purification by affinity chromatography [116]. In addition, tags can also protect against proteolysis or increase solubility (e.g., maltose-binding protein tags), ensuring better protein recovery [113]. Cleavage sites between the tag and recombinant protein allow tag removal post-purification. Co-expression of molecular **chaperones** (e.g., DsbC in *E. coli* SHuffle [135]) can prevent misfolding and IB formation and increase solubility. This approach has been successfully applied in *K. phaffii*, where chaperone coexpression increased human LYS (by 1.5-fold) and bovine lactoferrin (by 4.3-fold) yields [90]. In addition, **proteolytic degradation** by host proteases reduces the yield and quality of recombinant proteins, e.g., for structurally simpler proteins without protective features such as OVA [91]. This could be mitigated by using protease-deficient strains such as *E. coli* BL21 (with no *ion* or *OmpT* proteases), optimizing the pH and temperature, or using protease inhibitors [113].

### Bioproduction of recombinant egg proteins

**Fig. 2:**
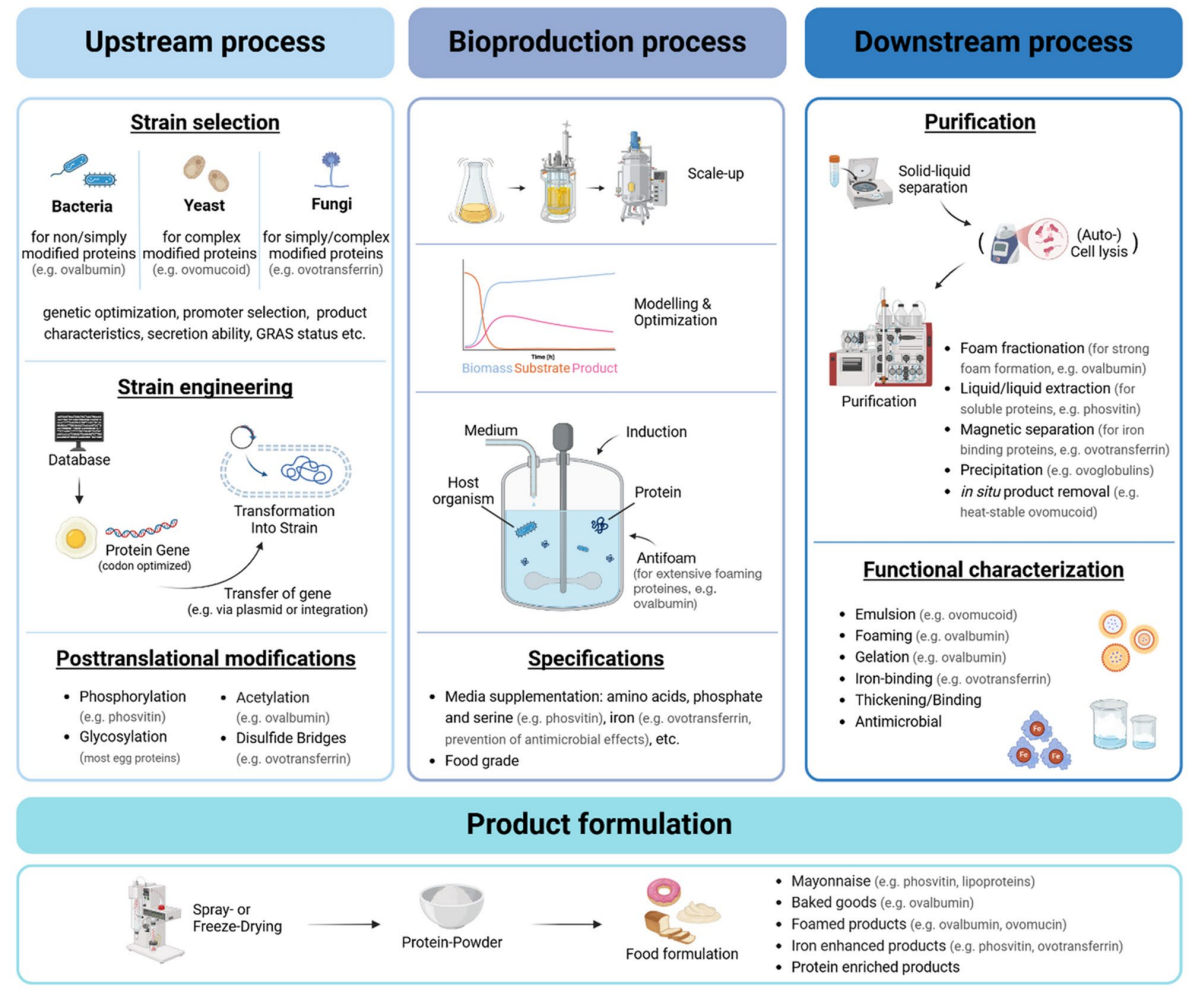
Scheme for a generalized PF production process for recombinant egg proteins (Created in BioRender. Beck, F. (2025) https://BioRender.com/arypnbf)

Recombinant egg protein production is still at the stage of current research and development. Laboratory-scale and preindustrial production processes have not been established and evaluated regarding their key performance indicators and economic viability. Each egg protein presents unique challenges due to its specific characteristics and thus special requirements to the production process, such as its molecular weight (up to 8300 kDa for OVN), AA composition (e.g., phosvitin contains 123 serines out of 216 AAs), PTMs (e.g. glycosylation in ovalbumin, phosphorylation in phosvitin) and structural features such as disulfide bridges (e.g. OVT with 15 disulfide bridges) (Table 1). An overview of the specific production scheme for recombinant egg proteins is given in Fig. 2.

A notable example of successful recombinant egg protein production is OVA synthesis in *Escherichia coli* Nissle 1917 (EcN) via an optimized, antibiotic-free approach developed by Liu et al. (2024). First, cultivation was performed in shaking flasks in TB medium at 30 °C and 220 rpm for 10 h with 0.1 mM IPTG. This was followed by high-density fermentation in a 3 L bioreactor using a glucose-based medium. A two-step feeding strategy (exponential and DO state positive feedback) combined with 0.5 mM IPTG induction ensured efficient OVA synthesis at 30 °C while maintaining the pH at 6.2–6.8 and the dissolved oxygen (DO) content at 35%. Key challenges, such as plasmid stability and metabolic overflow, were overcome by using the EcN endogenous plasmid and minimizing acetic acid production. DSP involves high-pressure homogenization (800 MPa, 5 min) for cell disruption and Ni-NTA affinity chromatography for purification. This resulted in a final yield of 3.70 g/L, the highest reported in *E. coli* (Table 2) [24]. Another example is the production of recombinant OVM by the EVERY Company (formerly known as Clara Foods Co., Daly City, CA, USA), which was reviewed under FDA GRAS (generally recognized as safe) Notification No. 967. The process uses *K. phaffii* strain GSD-1209, which secretes OVM directly into the fermentation medium. The strain was first cultured in BMGY media supplemented with glucose for two days and then transferred to BMMY media supplemented with methanol to induce recombinant protein production at 30 °C for two days. This is followed by extensive purification steps such as centrifugation, microfiltration, pH adjustment, and ultrafiltration, followed by affinity, ion exchange (pH 6.5–7.5), size-exclusion chromatography, and drying. The final product, a white to off-white powder, consists of > 75% protein, ≤ 20% carbohydrates, and < 0.1% fat. Maximum titers of 3.2 g/L after 168 h of cultivation have been reported (Table 2). The FDA confirmed the genetic stability of the strain and the absence of antibiotic resistance genes, raising no safety concerns, but required allergen labeling owing to the allergic nature of OVM [38, 93]. Moreover, in December 2024, Onego Bio informed the FDA about a GRAS notification for their Bioalbumen^®^, which is recombinant ovalbumin expressed with *Trichoderma reesei* [136]. These examples highlight the feasibility of recombinant egg protein production. However, significant challenges persist for the production process (Table 3) to achieve functional proteins and commercial viability. This includes, for example, the **foaming** ability of some egg proteins, such as OVA [90], which presents additional challenges, particularly in aerated bioreactors. Excessive foaming can lead to protein loss, operational inefficiencies, and contamination risks. The use of chemical antifoam agents or mechanical foam-control systems in bioreactors can mitigate these problems, but food-safe approaches must be prioritized (e.g., dimethicone (GRAS) status [123])) [122].

Despite advances in upstream fermentation strategies, **DSP** remains a significant cost barrier in recombinant egg protein production, often accounting for up to 80% of the total process costs [137]. Many DSP techniques, such as chromatographic methods, are less suitable for inexpensive bulk food protein purification and food-grade purity requirements. The choice of purification method depends on the characteristics of the protein (e.g., molecular weight, solubility, pI, etc.) and the production scale. The laboratory-scale method prioritizes high purity, whereas the industrial-scale method focuses on maximum recovery, efficiency and cost reduction [22]. In the laboratory, techniques such as **anion exchange chromatography** have been successfully applied to natural egg proteins, such as phosvitin (92.6% purity and 35.4% recovery based on total yolk phosvitin). Still, their high operating costs limit industrial scalability [61]. To address these challenges, alternative purification methods are being developed. Non-chromatographic methods, such as **precipitation techniques**, are among the simplest and most cost-effective methods for initial protein isolation. Common methods include salting-out with ammonium sulfate (e.g., OVT with > 83% yield and 85% purity while maintaining protein bioactivity [129]), organic solvent precipitation, and isoelectric point (pI) precipitation by adjusting the pH to the pI of the protein (e.g., OVA with 95.1% purity) [124]. However, precipitation methods often require additional desalting steps, such as dialysis, and may risk protein denaturation if not optimized properly [138]. Other more sustainable single step purification methods are **adsorption-based** techniques, already successfully applied for egg white LYS (94.3% recovery of total egg white [125]), and **aqueous two-phase systems**, applied for native LYS (99% recovery of LYS in “ionic-liquid-based aqueous biphasic systems” [127]), avidin (92% recovery [126]) and OVA (65% recovery [139])). **Magnetic separation** may offer a viable purification approach by utilizing the natural affinity of proteins to metal ions, e.g., OVT and phosvitin. Recent advances in magnetic purification systems have introduced a novel alternative for purifying iron-binding proteins such as lactoferrin, which could be adapted for egg proteins [128]. Krolitzki et al. (2024) reported a sequential process that combines ultrafiltration and magnetic separation, using iron oxide nanoparticles (BIONs) for cost-effective purification of lactoferrin (60% yield and 78% purity) [140]. Moreover, **foam fractionation** is a continuous process for the purification of products such as OVA due to its high foaming properties. In this process, proteins adhere to the surfaces of rising bubbles via their hydrophobic regions, are transported upwards with the bubbles and then collected. Ideally, the foam itself can be used directly as an end product without the need for further purification steps [141]. In summary, while chromatographic techniques remain essential for achieving high purity in research applications, methods such as magnetic separation and precipitation could provide scalable, eco-friendly, and cost-effective alternatives for industrial production. Another challenge is the maintenance of **protein stability and functionality** during production, storage, and distribution. Critical factors such as pH, ionic strength, temperature, and proteolytic degradation must be carefully managed to ensure product integrity. Long-term stability must be evaluated and guaranteed for post-production processes (e.g., transport and storage) and maintaining functional properties in food matrices [130]. In addition to the technical and biochemical challenges discussed, recombinant egg protein production faces hurdles regarding regulatory approval, consumer acceptance, and economic feasibility (cf. Section “Future perspectives”).

### Post-translational processing and modifications of protein functions

A major obstacle in the production of recombinant egg proteins is the incorporation and replication of PTMs (Table 3). They might be relevant for maintaining the techno-functional and bioactive properties of proteins. It is unclear whether PTMs are necessary for the techno-functionality of egg proteins in food applications. Based on current literature data, *E. coli* remains the most efficient host for REP production and will, therefore, be prioritized in this chapter.

For example, **phosphorylation** can introduce or enhance desirable properties in proteins, including improved solubility and thermal stability and increased emulsifying, foaming, and gelling abilities [130]. Phosvitin, a highly phosphorylated yolk protein with up to 90% of its serine residues modified, makes it particularly challenging to replicate in microbial systems [61]. Achieving phosphorylation is complex, as in the case of *E. coli*, this organism is deficient in serine/threonine/tyrosine protein kinases to produce eukaryotic phosphoproteins [113]. Engineered *E. coli* strains, such as *E. coli* B95(DE3) ΔA ΔfabR ΔserB, have been developed to incorporate phosphoserine site-specifically at the UAG codons via genetic code expansion without constructing prematurely truncated proteins [103, 117]. However, this system is currently limited to research settings and is not yet focused on large-scale production. In addition, deletion of the *serB* gene, which encodes the phosphoserine phosphatase responsible for removing phosphate groups from serines, increases phosphoserine synthesis (Δ*serB*) [114]. Media optimization with serine and phosphate supplementation may be needed to prevent nutrient depletion and ensure high-yield synthesis, e.g., for phosvitin, which is highly phosphorylated. In addition, in vitro enzymatic processing by using kinases (e.g., mitogen-activated protein kinase) and ATP as phosphate donors can be used to phosphorylate the specific sites on the recombinant protein [142]. Another relevant PTM is **disulfide bond formation**, which is a critical process to ensure correct protein folding and the functionality of especially cysteine-rich egg proteins, such as OVT and OVM (Table 2) [122]. Chaperone coexpression systems (e.g., DsbC) in *E. coli* can facilitate disulfide bridge formation and folding. Genetically engineered strains such as Origami™ 2, BL21trxB (DE3), and SHuffle™ (Express), which harbor *trxB* and *gor* mutations, allow for increased cytoplasmic disulfide bond formation, supporting the correct folding of complex egg proteins. Specialized strains such as Rosetta-gami and Novagen’s origami, which carry di-sulfide-enhancing mutations and rare tRNA expression systems to mitigate codon bias, help to improve the folding of recombinant proteins with complex di-sulfide structures, such as those found in egg proteins [90, 113, 118]. Moreover, all major egg proteins are naturally **glycosylated.** However, glycosylation is rare in *E. coli* because of the lack of eukaryotic glycosylation machinery [113]. A bacterial strain with a *ΔwaaL* mutation was established for successful glycosylation, e.g., the *E. coli* W3110 mutant CLM24 [121]. Moreover, in vitro N-glycosylation can be catalyzed by N-glycosyltransferase, which forms a glycosidic bond between an oligosaccharide donor and the asparagine residue [143]. Yeast-based expression systems such as *K. phaffii* offer significant advantages by performing N-glycosylation, although the glycosylation patterns may differ from those of avian proteins, potentially impacting protein functionality. To address this, alternative strains, such as *K. phaffii* GlycoSwitch^®^ strains (BioGrammatics, Carlsbad, CA), have been genetically modified to produce human-like glycosylation patterns [119, 120]. In general, PTMs increase protein solubility and destabilize the unfolded state, which would otherwise require a refolding step in the DSP, ultimately reducing costs [145]. All three PTMs appear in the native egg white protein OVA (Table 1), which is critical for its structural stability. For example, for efficient secretion from *K. phaffii*, N-glycosylation at Asn-292 of OVA may be required [109]. However, integrating multiple PTMs within a single host, especially in bacterial systems, has yet to fully develop, requiring either optimized co-expression strategies or alternative post-expression modification approaches. Producing recombinant egg proteins with nature-identical PTMs through targeted host engineering and process optimization is challenging but feasible. Nevertheless, the relevance of these PTMs for food applications remains uncertain.

## Future perspectives

The recombinant production of egg proteins is still in its infancy. The need for strain optimization and establishing a bioprocess that is adapted to meet the requirements of that specific product category remains crucial to reaching economically viable product titers of approximately 50 g/L for recombinant egg proteins, as stated by Nielsen et al. (2024) [89]. A thorough evaluation of economic metrics, including cost of goods sold, capital and operating expenditures, and projected return on investment, alongside a comprehensive commercial valuation, is essential to establishing the feasibility and market competitiveness of recombinant egg protein processes. However, a full techno-economic analysis and detailed market assessment lie beyond the scope of this study and will be addressed in future research. Several egg proteins naturally present in low concentrations exhibit strong functional properties (e.g., emulsifying, foaming, and iron-binding) and are prospectively relevant for commercial applications. This positions them as attractive, high-value functional ingredients. Furthermore, this characteristic could enable recombinant egg proteins to enter the market ahead of bulk protein alternatives, as they fulfill specific functional roles without needing mass production to meet overall protein demand. OVA, particularly in its dephosphorylated form, has thus far been expressed by several research groups, with the highest titer achieved for recombinant egg proteins (3.7 g/L in *E. coli* [24]). OVT requires correct disulfide bond formation and is particularly interesting since it can provide bioavailable iron due to its iron-binding ability. Phosvitin presents challenges due to its high degree of phosphorylated serines (90%). The hypothesis of whether PTMs are necessary for the required techno-functional properties of recombinant egg proteins in food applications remains open and is at the current research stage. Microbial protein secretion systems are most likely required since the total mass of intracellular recombinant proteins, such as IBs, is limited by biomass concentration. Since the protein product is intended to be used in food applications as a high-volume, low-value ingredient, space-time yields and titers of the final product must be significantly greater than those of proteins produced for the pharmaceutical industry. The DSP needs to meet food safety requirements while also maintaining a short, ideally single-step, cost-effective workflow to minimize losses and simplify large-scale production. Magnetic purification presents a scalable and efficient DSP alternative, particularly for iron-binding proteins such as OVT. In addition, an alternative approach to reduce DSP costs is to use food-grade, nonpathogenic microorganisms that secrete the target protein directly into the medium. Moreover, depending on the complexity of the downstream DSP and the extent of IBs or intracellular protein formation, simplifying water removal (98%) and, thus, energy costs may be advantageous. If the same regulatory conditions as those for food fermentation were applied, where the full lysate is consumed, DSP would not be needed, making the process significantly less expensive. However, regulatory pathways must be established before such methods can be widely adopted. Advances in synthetic biology and (AI-driven) process optimization could be used to improve host performance and production efficiency by tailoring genetic pathways that support efficient protein expression and PTM incorporation, if necessary, or implementing optimal feeding regimes. In addition to bioprocessing, integrating renewable energy sources is crucial for making energy-demanding recombinant protein production economically viable and ecologically sustainable [92]. In the long term, recombinant proteins could be used to develop food applications, and techno-functional protein profiles and nutritional values could be engineered to meet functional or dietary requirements. Regulatory issues for recombinant food proteins in the European Union remain challenging. Moreover, public acceptance is crucial to the success of these products, but the outlook is favorable as consumers continue to seek alternative products. Education of the public on the production process and the environmental and ethical benefits of these proteins is necessary to build trust. Transparent labeling could reduce consumer concerns, particularly regarding GMOs. Establishing clear regulatory frameworks and comprehensive guidelines for recombinant proteins will be fundamental in facilitating the commercialization of recombinant egg proteins.

## Conclusion

Population growth, increasing wealth, and environmental concerns have increased the global consumer demand for sustainable animal protein. PF, a modern branch of industrial biotechnology, is expected to contribute to protein transformation as an environmentally friendly protein production method. Compared with conventional agriculture, increasing CO_2eq_ penalties for environmentally unbeneficial practices issued by the European Union are expected to increase the economic importance of technologies with a reduced CO_2eq_ emission profile per gram of protein. Furthermore, with the most recent spike in egg prices in the United States of America and increasing trends toward deglobalization through tariffs and protectionist governments, an additional source of food protein is essential to ensure the resilience, flexibility, and security of national food systems. Egg proteins are the fifth most consumed source of protein in the human diet and are valued for their nutritional and techno-functional properties in food applications, e.g., in baked goods. Based on the current literature, *E. coli* is recognized as the most efficient host for recombinant egg protein production, whereas *K. phaffii* is preferred for proteins requiring extensive PTMs. While six egg proteins (OVA, OVT, OVM, avidin, α-livetin, and egg white LYS) have been successfully produced recombinantly, current production remains inefficient and limited to laboratory-scale processes. However, current expression titers (Table 2) remain below the levels required for economic viability (titers of approximately 50 g/L) in large-scale food protein production [89]. Given the low technology readiness level of recombinant egg protein production, all areas of the bioprocess chain (upstream, bioproduction, and DSPs) require further improvements and research to increase protein titers, space-time yields, and production rates while maintaining protein functionality. Furthermore, food-safe and cost-reduced DSPs are needed to achieve commercial viability. Despite these challenges, PF remains a promising technology that broadens the spectrum of alternative protein manufacturing technologies. As a sustainable alternative to traditional animal agriculture, PF may provide a valuable tool to reduce animal suffering and environmental impacts and increase the robustness of the food system on a global scale.

## Electronic supplementary material

Below is the link to the electronic supplementary material.

Supplementary Material 1

Supplementary Material 2

## Data Availability

No datasets were generated or analysed during the current study.

## Abbreviations

AA: Amino acid
AP: Alternative protein
*B. subtilis*: *Bacillus subtilis*
CO_2_: Carbon dioxide
DSP: Downstream process
*E. coli*: *Escherichia coli*
EY: Egg yolk
EYG: Egg yolk granule
EYP: Egg yolk plasma
FDA: Food and Drug Administration
GHG: Greenhouse gas
GMOs: Genetically modified organisms
GRAS: Generally recognized as safe
HDL: High-density lipoprotein
IB: Inclusion body
IPTG: Isopropyl-ß-D-thiogalactopyranoside
*K. phaffii*: *Komagataella phaffii*
LDL: Low-density lipoprotein
LYS: Lysozyme
OVA: Ovalbumin
OVM: Ovomucoid
OVN: Ovomucin
OVT: Ovotransferrin
pI: Isoelectric point
PTM: Posttranslational modification
PF: Precision fermentation
T_d_: Denaturation Temperature
