# Supplementary material for: Scientific and technological challenges of recombinant egg protein production
Source: BMC Biotechnol. 2025 Jul 2;25:65. doi: 10.1186/s12896-025-01002-3 (PMC12224593; doi:10.1186/s12896-025-01002-3)
Supplement: Supplementary file 1 — Supplementary Material 1 [file 12896_2025_1002_MOESM1_ESM.docx]

**Additional file 1**

**Table 1:** Techno-functional, bioactive, and additional egg white and yolk protein properties

| **Protein** | **Techno-functions** | **Bio-functions** | **Additional functions** | **References*** |
| --- | --- | --- | --- | --- |
| **Major egg white proteins** | | | | |
| **Ovalbumin** | - Emulsion - Foaming - Gelling | - Antimicrobial - Antioxidative - Anticancer | n.d. | (Li et al. 2022; Sarantidi et al. 2023) |
| **Ovotransferrin** | n.d. | - Antimicrobial - Antifungal - Antiviral - Immunomodulatory effects - Anticancer - Antioxidative (weak) | - Binding and transport of iron metals (Fe, Cu or Zn) | (Nadeau, Falick, and Woodworth 1996; Wu and Acero-Lopez 2012) |
| **Ovomucoid** | - Emulsion - Foaming | - Antioxidative - Anticancer | n.d. | (Dupuis et al. 2023; Réhault-Godbert, Guyot, and Nys 2019) |
| **Ovomucin** | - Foaming - Foam stability - Emulsion | - Antimicrobial - Anticancer - Antiviral - Immunomodulatory effects | - Gel-like structure of egg white - Thinning of egg white during storage | (Omana, Wang, and Wu 2010; Sarantidi et al. 2023) |
| **Lysozyme** | - Foam stabilizer for wine | - Antimicrobial - Immunomodulatory effects - Anticancer - Antiviral - Anti-inflammatory | n.d. | (Li et al. 2022; Sarantidi et al. 2023; Guha, Majumder, and Mine 2019) |

| **Major egg yolk proteins** | | | | |
| --- | --- | --- | --- | --- |
| **LDL** | - Emulsion (apolipoproteins) | n.d. | n.d. | (M. Anton et al. 2003) |
| **HDL** | - Emulsion | - Antioxidant - Antimicrobial | n.d. |  |
| **Livetin** | - Emulsion - Foaming | - Immunomodulatory effects | n.d. | (Chalamaiah et al. 2017; Sarantidi et al. 2023) |
| **Phosvitin** | - Emulsion stabilizer | - Antioxidant - Antimicrobial - Anti-inflammatory - Anticancer - Promotion of bone health - Immunomodulatory effects | - Strong metal-affinity (especially iron) | (Marc Anton, Castellani, and Guérin-Dubiard 2007; Oladimeji and Gebhardt 2023; Sarantidi et al. 2023; Sattar Khan et al. 2000) |
| **Minor egg white proteins** | | | | |
| **Ovoglobulin** | - Foaming | n.d. | n.d. | (Guha, Majumder, and Mine 2019) |
| **Cystatin** | n.d. | - Antimicrobial - Anticancer - Immunomodulatory effects | n.d. | (Sarantidi et al. 2023; Wesierska et al. 2005) |
| **Ovomacroglobulin** | n.d. | - Antibacterial - Anti-inflammatory - Protease inhibitor | n.d. | (Kitamoto, Nakashima, and Ikai 1982) |
| **Avidin** | n.d. | - Antimicrobial - Anticancer - Insecticidal | - High affinity and specificity for biotin binding, which is used for e.g. molecular recognition | (Li et al. 2022; Sarantidi et al. 2023) |
| **Ovoflavin** | n.d. | n.d. | - Ability to bind to riboflavin or vitamin B2 | (Guha, Majumder, and Mine 2019) |

‘n.d.‘ represents not defined, *****see list of references in the main file
