# Supplementary material for: Scientific and technological challenges of recombinant egg protein production
Source: BMC Biotechnol. 2025 Jul 2;25:65. doi: 10.1186/s12896-025-01002-3 (PMC12224593; doi:10.1186/s12896-025-01002-3)
Supplement: Supplementary file 2 — Supplementary Material 2 [file 12896_2025_1002_MOESM2_ESM.docx]

**Additional file 2**

**Table 1:** Differences in bacteria, yeast and fungi expression systems for recombinant egg protein production

|  | **Bacteria**  (e.g., *E. coli*) | **Yeast**  (e.g., *K. phaffii*) | **Fungi**  (e.g., *T. reesei*) | **Reference*** |
| --- | --- | --- | --- | --- |
| **Expression system** | - Positive and negative regulated expression systems - Inducible system (e.g., IPTG) - large variation of vectors with tags, signal peptides, and protease sites - Easy genetic manipulation - Expression of larger proteins - Genetic stability of host and plasmid (both segregationally and structural) - Acetate formation causing cell toxicity | - Easy genetical manipulation - Strong promoters (AOX1, GAP) - Inducible system - Few tags, host strains, and selection markers available - Available methanol-based systems (oxidative stress, protein degradation) | - Complex regulatory systems - Synthetic promoters | (Dupuis et al. 2023; Meyer and Schmidhalter 2014; Schmoll and Dattenböck 2016; Sambrook, Fritsch, and Maniatis 2012) |
| **Productivity (rec. proteins)** | - Good productivity (up to 3.7 g/L ovalbumin) - Low for complex proteins (up to 4 mg/L ovotransferrin) | - Good productivity (up to 3.5 g/L lysozym) - Good for complex proteins (up to 97 mg/L ovotransferrin) | - Good productivity (up to 2 g/L ovalbumin) | (Table 2) |
| **Doubling time, Cell density** | - Short doubling time (30 min) - High cell density (160-200 g/L dcw) | - Moderate doubling time (1.5-2 h) - Very high cell density (up to 150 g/L dcw) | - Long doubling time (~ 2-3 h) - Low cell density (up to ~ 30 g/L dcw) | (Dupuis 2024; Sambrook, Fritsch, and Maniatis 2012) |
| **Secretion, product location, solubility issues, proteolysis** | - Mostly intracellular protein production requires lysis - IBs formation (possible) - /+ Solubility issues concomitant to high level recombinant protein expression - Secretion challenges - Proteases should be removed | - Extracellular protein production - IBs free, good solubility - Secretion of recombinant proteins possible - Some proteolytic degradation possible | - Extracellular protein production - IBs free, good solubility - Efficient secretion - Protease activity (protein degradation possible) | (Meyer and Schmidhalter 2014; Sambrook, Fritsch, and Maniatis 2012; Dupuis 2024)  (Table 2) |
| **PTMs, protein folding** | - No natural PTMs (potential need of specific e.g., phosphorylation systems (*E. coli* B95(DE3) ΔA ΔfabR ΔserB)) - Large proteins may fold improperly (need of chaperones (e.g. Shuffle™ (Express)) | - Many PTMs, including disulfide bridge formation, glycosylation, and phosphorylation - Improved folding of large proteins - Proteins can be core glycosylated (high-mannose type) - Inability to perform complex PTMs | - Many PTMs, including disulfide bonds and glycosylation - Good protein folding - Different glycosylated patterns possible (e.g. hyperglycosylation) | (Knychala et al. 2024; Meyer and Schmidhalter 2014; Sambrook, Fritsch, and Maniatis 2012)  (Table 3) |
| **System robustness, endotoxins** | - Robust system - Endotoxin production possible | - Good robustness - Tolerates harsh conditions - No endotoxins | - Robust system - No endotoxin production (aflatoxin) | (Meyer and Schmidhalter 2014) |
| **Cost, equipment, cell maintenance** | - Low-cost and simple media (e.g. glucose) - Low-cost production - Easy cell maintenance - Intensive CAPEX (e.g., for bioreactor) | | - Cheap media (e.g. lignocellulose) - Requires more specialized equipment - Intensive CAPEX (e.g., for bioreactor) - Requires more cell maintenance | (Dupuis et al. 2023; Sambrook, Fritsch, and Maniatis 2012) |
| **Scalability** | Research is needed for efficient up-scaling of recombinant egg protein production | | |  |

*****see list of references in the main file
